# Delayed cerebrospinal fluid leak (CSFL) occurring 21 days post-lumbar unilateral biportal endoscopic surgery: A case report

**DOI:** 10.1097/MD.0000000000046858

**Published:** 2026-01-09

**Authors:** Xinjian Feng, Jintao Shi, Xueping Huang

**Affiliations:** a Department of Neurospinal Surgery, Luzhou People’s Hospital, Luzhou City, China.

**Keywords:** delayed cerebrospinal fluid leak, dural tear, lumbar drainage, unilateral biportal endoscopic spine surgery

## Abstract

**Rationale:**

While minimally invasive endoscopic surgery has emerged as the primary approach for managing lumbar degeneration, cerebrospinal fluid leakage (CSFL) remains a common complication in traditional open spinal procedures. However, the literature documenting CSFL occurrence in minimally invasive endoscopic interventions is scarce, with delayed-onset cases (beyond 20 days postoperatively) representing an exceptionally rare clinical entity.

**Patient concerns:**

We describe the case of a 60-year-old man who underwent unilateral transforaminal endoscopic spine surgery. On the 21st postoperative day, a cystic mass developed at the surgical site.

**Diagnoses:**

Delayed postoperative CSFL was diagnosed.

**Interventions:**

The patient underwent a sequence of interventions: initial pressure bandaging and skin suturing, followed by lumbar cistern drainage and open dural repair surgery (which failed), and ultimately a successful 14-day course of continuous lumbar drainage. Post-drainage, he received core lumbar muscle training for rehabilitation.

**Outcomes:**

The continuous lumbar drainage ultimately resolved the CSFL after 14 days, with drainage volume peaks noted on days 7 and 10. At recovery, the patient resumed daily activities; however, he retained a residual reduction in right ankle dorsiflexion muscle strength (grade 3/5).

**Lessons:**

Managing delayed CSFL is complex. While continuous lumbar drainage can be an effective definitive treatment, it may require an extended duration. This case underscores the importance of vigilant postoperative monitoring for delayed complications.

## 1. Background

Surgery for lumbar degeneration can lead to several complications, with cerebrospinal fluid leakage being the most prevalent.^[1]^ Beyond established risk factors (age, indication, and procedure type), the incidence of intraoperative dural tears with subsequent cerebrospinal fluid leakage (CSFL) may be influenced by systemic comorbidities (diabetes, hypertension, obesity) and behavioral factors (notably smoking).^[2,3]^ It is usually detected within the first week after surgery, during the patient’s follow-up. This issue often stems from the unnoticed and unrepaired dural tears that occur during minimally invasive endoscopic procedures. There are almost no reports of a delayed CSFL after endoscopic surgery. In contrast, delayed CSFL following traditional surgery is quite uncommon, with an incidence rate of 0.59% to 0.85%.^[4,5]^

In this report, we describe a patient who developed delayed CSFL following unilateral biportal endoscopic (UBE) spine surgery of the lumbar spine. Our cases illustrate the complete progression of the disease in treated patients.

## 2. Case presentation

**Figure 1.**
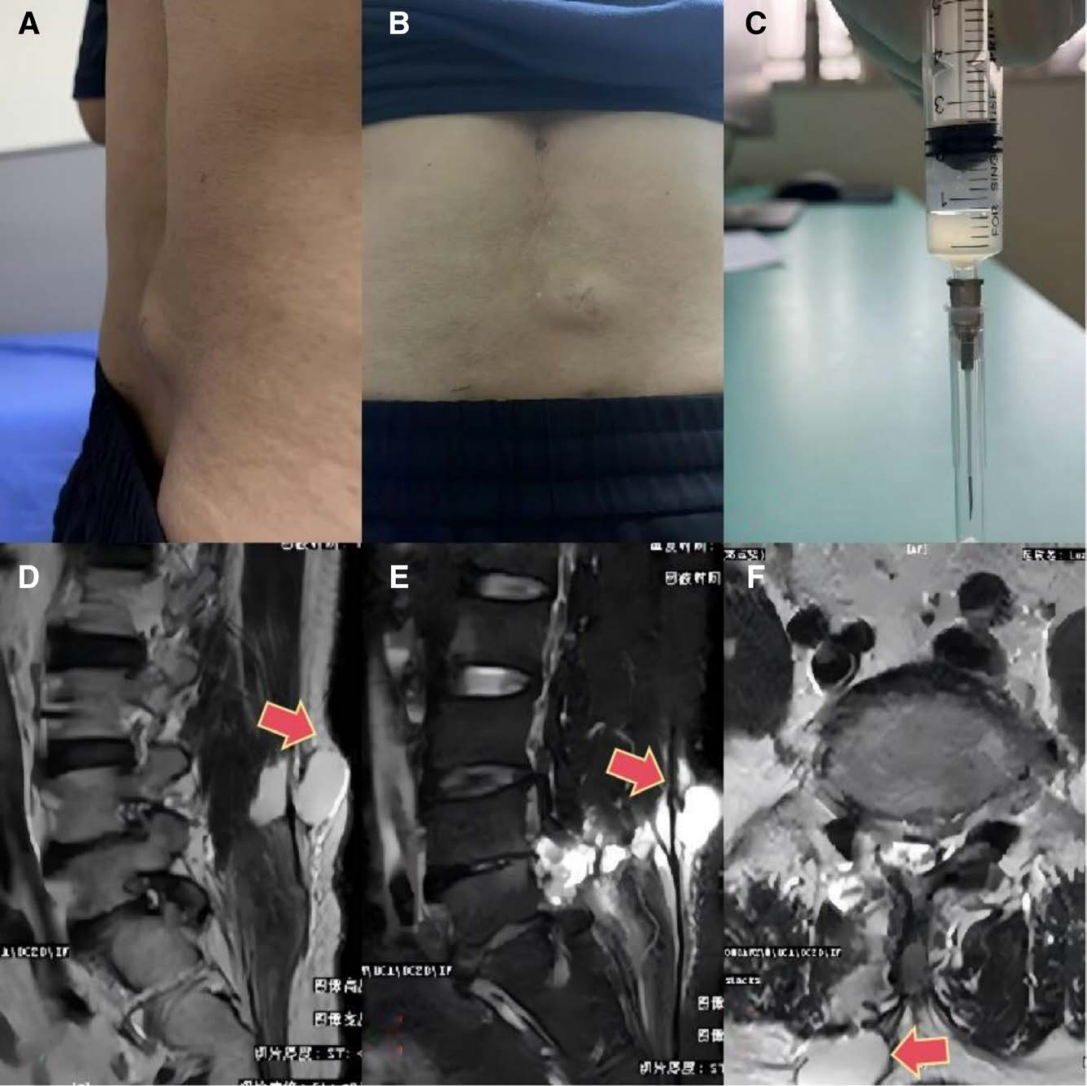
(A, B): Cystic masses were observed in the skin at the surgical area of the lumbar following UBE procedure. (C): Analysis of the puncture fluid from the lumbar mass revealed its properties.(D, E, F): MRI imaging demonstrateed subcutaneous accumulation of cerebrospinal fluid (CSF) at the L4-5 segments. The effusion was contiguous with the spinal canal. MRI = magnetic resonance imaging, UBE = unilateral transforaminal endoscopic spine surgery.

The patient was a 60-year-old man who presented to the neurospinal surgery department with a lumbar mass in the skin of the lumbar surgery area a week ago. He had undergone UBE surgery for lumbar disc herniation 28 days prior and was confirmed to have a delayed CSFL by puncture fluid and magnetic resonance imaging (MRI) (Fig. 1).

## 3. Non-open dural tear repair

**Figure 2.**
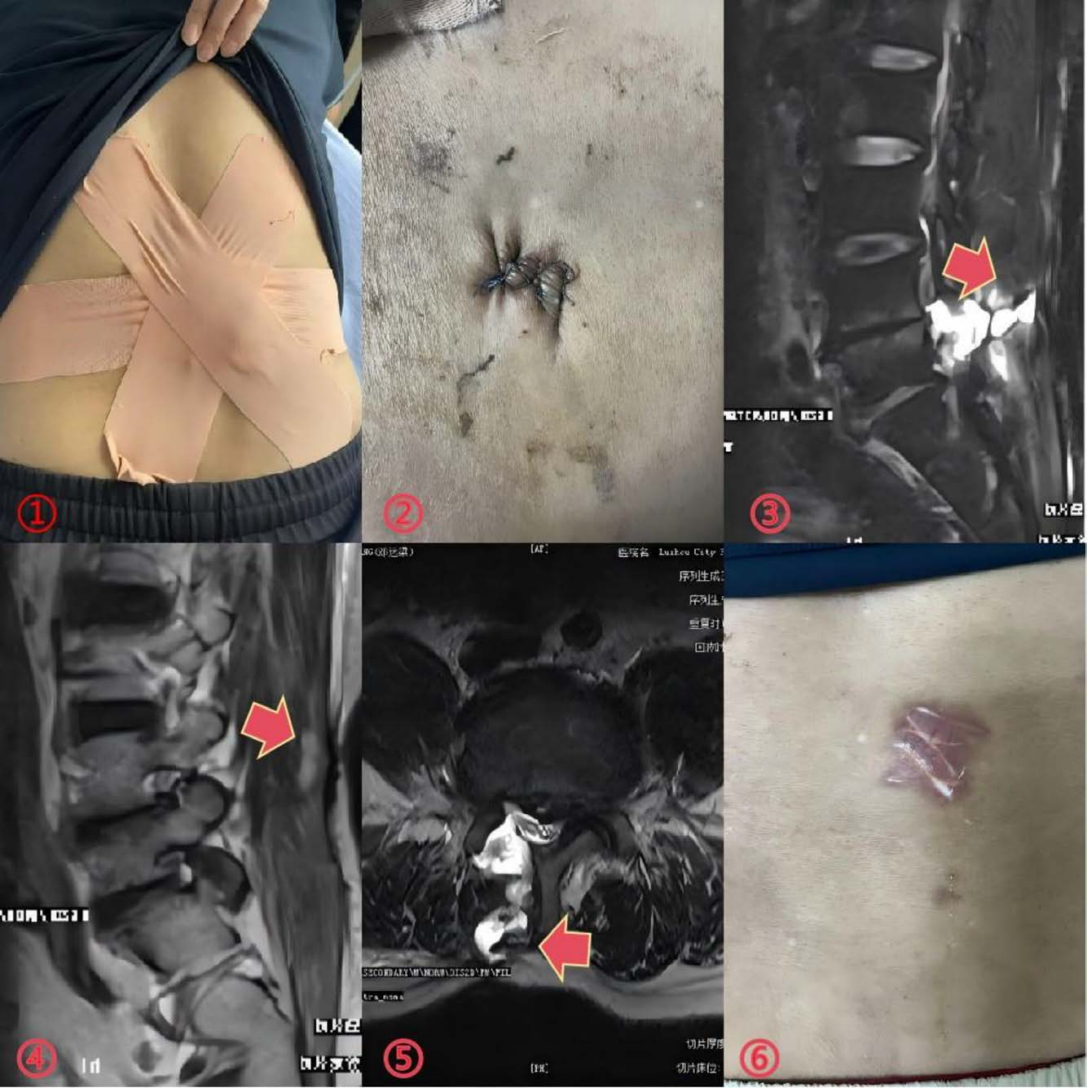
①: The lumbar mass was packed with gauze, and a pressure dressing was applied to manage the swelling and prevented further complications. ②: An attempt was made to close the subcutaneous sinus ostium through the skin, aiming to promote healing and prevented cerebrospinal fluid (CSF) leakage. ③④⑤: MRI revealed a reduction in subcutaneous effusion, indicating partial resolution of the fluid collection. However, the sinus remained patent. ⑥: CSF leakage was observed at the patient’s skin sutures.

Initially, we packed the mass with a large gauze pad and applied a compression bandage, advising the patient to rest in a supine position (Fig. 2(①)). One week later, the patient’s lumbar mass persisted. We attempted to close the subcutaneous leaks by using skin sutures (Fig. 2(②)). A week later, MRI revealed a decrease in subcutaneous cerebrospinal fluid (CSF) (Fig. 2(③-⑤)), and the patient was subsequently discharged.

**Figure 3.**
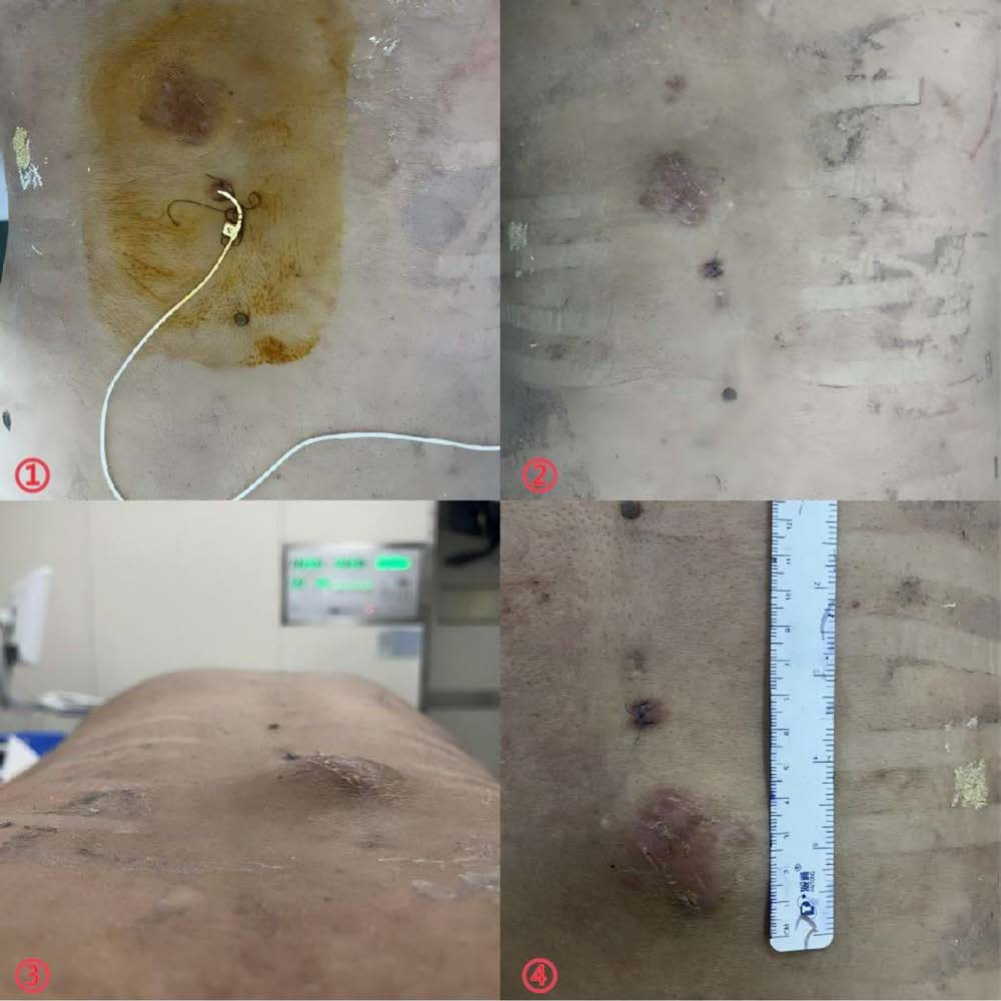
①②③④: After implanting lumbar cisternal drainage for a week, the drainage was not smooth and the skin healed, but the lumbar mass appeared again.

Ten days later, fluid leakage occurred in the patient’s skin sutures (Fig. 2(⑥)), and lumbar cistern drainage was urgently performed (Fig. 3(①)). After a week of drainage, fluid leakage ceased and the wound healed (Fig. 3(②)). The drainage tube was removed due to inadequate drainage, but the lumbar mass reappeared (Fig. 3(③-④)).

## 4. Open dural tear repair

**Figure 4.**
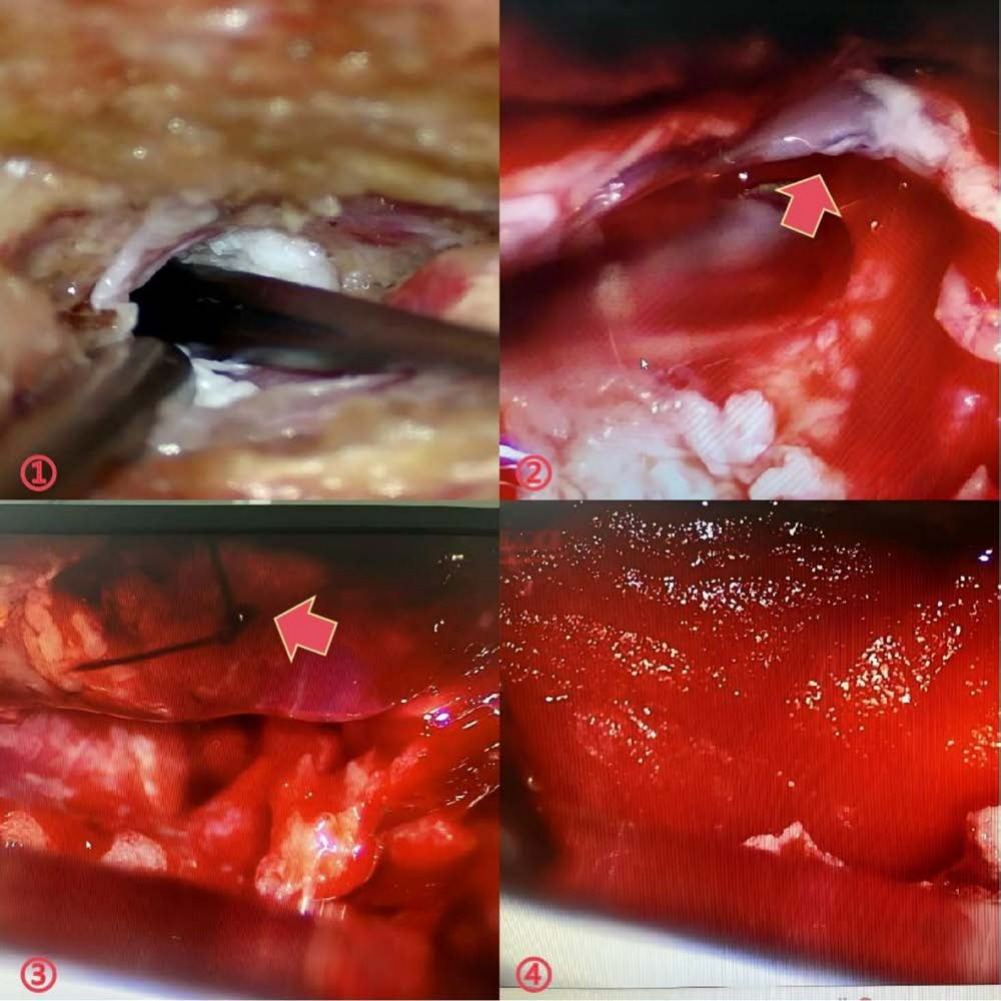
①: Subcutaneous sinus. ②: Dural tear defect(>10 mm), full-thickness. ③: We chose to cover the defect with muscle and suture it directly. Fibrin glue was then added. ④ Implanted dural patches.

The patient returned to the operating room for open dural tear repair. Upon reaching the desired level, a sinus ostium was discovered beneath the skin, from which a substantial amount of CSF flowed (Fig. 4(①)). After complete drainage of the CSF, we identified a dural tear (>10 mm) with full-thickness involvement (Fig. 4(②)). We opted to cover the defect with muscle and suture it directly (Fig. 4(③)). Subsequently, fibrin glue was injected and a dural patch was implanted (Fig. 4(④)). A controlled Valsalva maneuver (peak pressure: 20–30 cmH₂O) was performed after repair, demonstrating no CSF egress at the suture site. Following irrigation, thorough exploration of the wound revealed no bleeding or other structural damage, and a lumbar drain was subsequently placed. The operation was performed without any complications.

Following the surgery, the patient remained in the supine position. On postoperative day post-operation, the patient was advised not to get out of bed. Initially, the volume of fluid drained from the lumbar drain increased, followed by daily fluctuations in drainage output ranging between 50 and 160 mL. Two distinct peaks in drainage volume were observed on Day 7 and Day 10 during the drainage course. On the 13th and 14th days post-operation, the patient’s CSF drainage was recorded at 5 ml and 8 ml, respectively. We then removed the drainage tube and sutured the wound securely. Upon identification of CSF leakage through the skin sinus, comprehensive microbiological workup (including CSF cultures and biochemical analysis) was performed, showing no evidence of infection. However, considering the need to prevent intracranial infection, prophylactic intravenous ceftriaxone (2 g daily) was administered continuously until complete resolution of the CSF leak.

## 5. Functional training, follow-up, and outcomes

**Figure 5.**
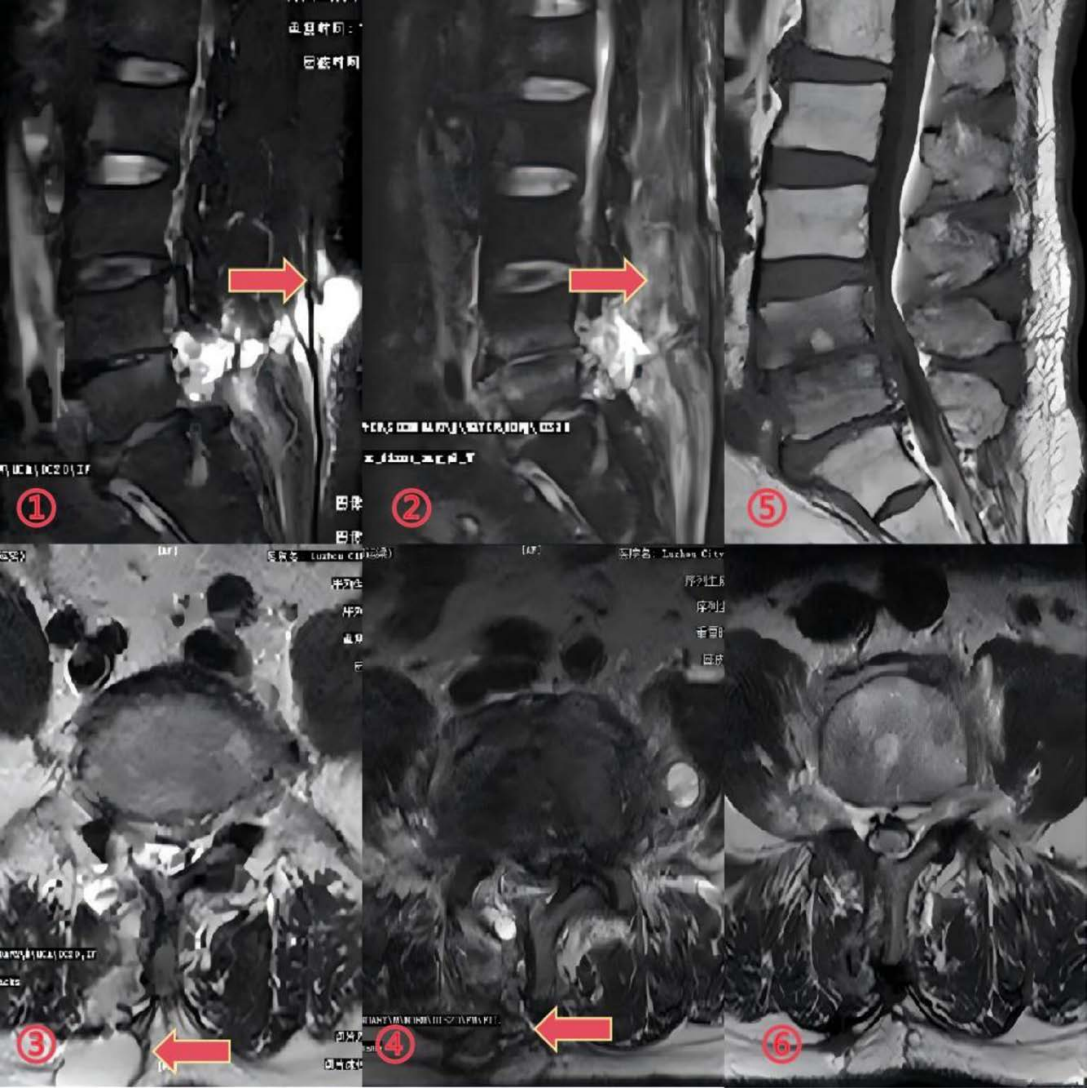
①③: Preoperative MRI examinations were performed prior to lumbar drainage. ②④: One week after lumbar drainage, follow-up MRI examinations confirmed resolution of the cerebrospinal fluid (CSF) leak. ⑤⑥: Three months postoperatively, MRI examinations were repeated to assess long-term outcomes. MRI = magnetic resonance imaging.

**Figure 6.**
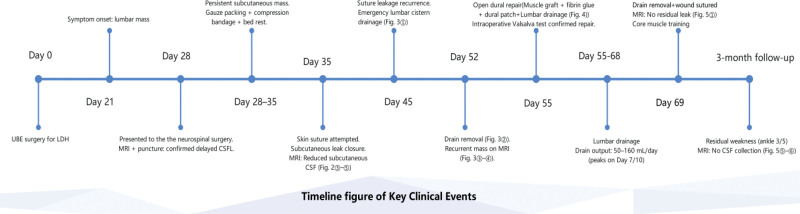
Timeline figure of key clinical events.

Subsequently, the patient ambulated but developed lower back pain and weakness. After 1 week of core muscle training, daily activities were gradually resumed. However, physical examination revealed right ankle dorsiflexion weakness (grade 3/5). MRI demonstrated controlled CSF leakage without evidence of neural compression or inflammatory changes (Fig. 5(②④)). Despite radiographic resolution of the subcutaneous sinus tract and CSF collections at 3-month follow-up (Fig. 5(⑤⑥)), the patient exhibited persistent right ankle dorsiflexion weakness (grade 3/5). For enhanced clarity of the clinical course, Figure 6 provides a detailed timeline of all critical events, from initial surgery through final follow-up. This visual summary complements the textual case description and may serve as a quick-reference guide for clinicians.

## 6. Discussion

This report presents a unique case of delayed CSFL after lumbar UBE surgery. The patient also had a lumbar mass accompanied by pain. MRI and fluid aspiration confirmed the presence of a CSFL, which was identified as a lumbar mass. During the open dural tear repair procedure, a significant amount of CSF was noted in the subcutaneous sinus opening. Upon reviewing the surgical video of this patient’s UBE procedure, we identified no accidental dural incision or abnormal fluid flow intraoperatively. Postoperative CSFL following UBE surgery, although rare, poses significant clinical challenges, particularly when presenting as a delayed complication.

Dural tear with CSFL represents one of the most frequent intraoperative complications in both open and endoscopic spine surgery, with comparable incidence rates.^[6,7]^ Endoscopic spine techniques encompass microendoscopic, uniportal endoscopic, and UBE. Over the past decade, UBE has garnered substantial clinical interest. Lv et al demonstrated that the dural tear rate in UBE decompression was comparable to those of conventional microscopic and uniportal endoscopic techniques, without statistically significant differences.^[8]^ In fact, the dural tear rate in UBE surgery is lower than that in microendoscopic techniques.^[9]^ This advantage may be attributed to the saline irrigation in UBE, which provides a clear surgical field and magnified visualization of adhesions between the dura mater and ligamentum flavum. Surgeons must meticulously dissect these structures before ligament removal to reduce dural injury risk. Such precision contributes to UBE’s capability of minimizing dural tears. However, compared to uniportal endoscopy, UBE carries a higher early-stage dural tear risk (4.8% vs 0.84%).^[10]^ Uniportal endoscopic techniques involve 2 approaches: interlaminar and transforaminal. Both the interlaminar approach and UBE are paraspinal techniques, with a reported dural tear rate of 2%.^[11]^ In contrast, the transforaminal approach demonstrates a lower dural tear rate (0.9%), typically with smaller tear sizes. Given its minimal skin incision and long lateral trajectory, even when dural tears occur, fibrin sealant alone often suffices, rendering the risk of clinically significant CSF leakage negligible.^[12]^

In our clinical practice, the endoscopic treatment of dural tears poses a significant challenge for physicians. Direct suture repair using nonabsorbable sutures is the traditional approach for the repair of durotomy.^[13,14]^ The decision to perform direct repair depends on factors such as the size and location of the rupture as well as whether the dura mater is completely or partially compromised.^[15]^ While some minor dural tears may not necessitate suturing, a tear exceeding 10 mm that remains unsutured during surgery significantly increases the risk of CSFL.^[16,17]^ However, if a dural tear is not visible and cannot be sutured directly, alternative repair methods should be considered. These methods may include the use of fascia or muscle, fibrin glue sealant, or commercial patch.^[18–20]^ CSFL identified during open surgery can typically be repaired immediately. However, in this case, the patient presented with a lumbar mass as a symptom, prompting the physician to opt for non-surgical treatments, such as local compression or suturing.^[21]^ Unfortunately, the suture was insufficient; therefore, the subcutaneous sinus opening was not closed, and finally, CSFL occurred through the skin. To mitigate the risk of intracranial infection, lumbar cistern drainage was performed. Although CSFL from the skin was subsequently controlled, the lumbar mass persisted. Finally, we had to choose an open patching.

After a comprehensive literature review, it appears that minimally invasive endoscopic surgery is associated with a lower incidence of CSFL, and may actually be the opposite.^[22,23]^ In a retrospective analysis of 3179 patients undergoing posterior lumbar surgery, Tang et al identified 4 independent risk factors for CSFL: type of disease, preoperative epidural steroid injections, multilevel procedures, and revision surgery.^[24]^ Notably, the CSFL incidence varied significantly by diagnosis degenerative spinal pathologies demonstrated the highest rate (33.3%), followed by spondylolisthesis (3.3%), lumbar spinal stenosis (2.5%), and lumbar disc herniation (0.7%). The patient in this case had an epidural injection of steroid hormones 2 days before UBE surgery. Although the torn dura mater was found during open repair surgery, its cause is not known. CSFLs are difficult to detect during endoscopic spinal surgery because of the continuous use of fluid irrigation.^[25]^ Furthermore, UBE surgery involves the creation of a tunnel beneath the muscle, which can lead to the separation of the muscle and lamina. However, this procedure may create more dead space.^[23]^ In cases of small dural tears, CSF may accumulate beneath the skin, potentially resulting in herniation through the skin. This may be the cause of delayed CSFL. Based on our clinical experience and literature evidence, We propose the following intraoperative strategies to minimize dead space and potentially mitigate delayed CSFLs: Precision preoperative localization and surgical pathway planning to maximally reduce unnecessary muscle dissection and laminectomy extent. Low-pressure irrigation system: Maintaining controlled irrigation pressure to decrease fluid retention in dead spaces while ensuring optimal visual clarity. Arthroscopy-assisted uniportal spinal surgery: As an innovative modification of UBE, arthroscopy-assisted uniportal spinal surgery provides a novel approach for lumbar degenerative disease treatment. This technique requires only a single 2-cm incision, demonstrating significantly reduced soft tissue trauma and neural retraction compared to conventional UBE.^[26]^

After the procedure, patients were instructed to rest in bed. Farshad^[27]^ et al published a randomized controlled trial involving 60 patients who were assigned to either 48 hours of bed rest (30 patients) or early postoperative ambulation (30 patients). No significant differences were observed in the occurrence of persistent postoperative CSFL. However, the bed rest group experienced a greater number of medical complications. On the second postoperative day, the patient disregarded this instruction and got out of bed, resulting in a significant increase in the volume of CSF drainage volume. The patient had to rest in bed and insist on lumbar drainage. The drainage volume temporarily decreased 7 days after the operation. This phenomenon is consistent with the views of Kim, who presented the biological sequence of events involved in the healing process of dural defects.^[17]^ Cain et al^[28]^ reported that fibroblastic bridging begins on day 6 post-injury, with complete healing occurring by day 10. After 14 days of drainage, the lumbar drainage tube was pulled out and reinforced, and the CSFL was cured.

Lumbar drainage directly reduces intrathecal pressure, creating a favorable gradient for dural healing. Studies have demonstrated that maintaining CSFL at 5 to 15 mL/h, significantly decreases the pressure at the leak site, promoting spontaneous sealing without revision surgery.^[29]^ In UBE procedures, where dural defects are often small and localized, this approach aligns with the minimally invasive philosophy by avoiding reoperation.^[15]^ Moreover, continuous drainage allows quantitative assessment of CSF production and quality, enabling early detection of complications, such as infection, subdural hematomas, cerebellar sag, and patient mobility limitations.^[30,31]^ We concluded that if we actively chose lumbar drainage at the beginning, a subsequent, cutaneous fistula might be avoided.

## 7. Conclusion

Delayed CSFL, typically attributed to arachnoid granule inflammation or delayed tissue necrosis, often demonstrates poor responsiveness to conservative management. Prompt open surgical dural repair should be actively considered in such cases. While UBE offers minimally invasive advantages, its potential for delayed CSFL warrants careful consideration. Our case highlights 3 critical clinical insights: Intraoperative decision-making should be guided by dural tear size, with lumbar drainage serving as an effective and reliable treatment for CSFL. Postoperative vigilance: Delayed CSFL may require prolonged drainage, emphasizing the need for standardized protocols. Future studies should prospectively compare delayed CSFL rates across endoscopic techniques and establish evidence-based management guidelines for delayed presentations.

## Acknowledgments

Thank you to everyone who participated in this report.

## Author contributions

**Conceptualization:** Jintao Shi.

**Software:** Jintao Shi.

**Writing – original draft:** Xueping Huang.

**Writing – review & editing:** Xinjian Feng, Xueping Huang.
